# Developing an online continuing professional development course for busy healthcare professionals: 12 tips for course developers

**DOI:** 10.15694/mep.2019.000170.1

**Published:** 2019-09-12

**Authors:** Elaine Mealey

**Affiliations:** 1 St George's University of London

**Keywords:** Health professionals, Continuing Professional Development (CPD), eLearning, online learning, short courses

## Abstract

This article was migrated. The article was marked as recommended.

Health professionals are finding it increasingly hard to attend Continuing Professional Development (CPD) that involve time away from work. Short CPD courses offered as eLearning can help meet the flexible needs of these busy professionals. Framework and guidelines for developing these courses is lacking. This article aims to provide readers with practical advice based on the author’s personal experiences and the literature available to help course developers construct an engaging course for this specific audience. Tips are offered from the initial scoping exercise, to developing the course, carefully considering the specific differentiation and technical support that is required for developing online learning activities and assessments compared to face to face CPD courses for a diverse range of learners.

## Introduction

Health professional registrants of the Health and Care Professional Council, General Medical Council, General Dental Council and Nursing and Midwifery Council must meet CPD requirements to remain registered. CPD activities need to be a mixture of learning activities relevant to current or future practice to keep skills and knowledge up to date, in order that health professionals are able to practise safely and effectively. Opportunities for education (CPD) after completing pre-registration training ‘vary depending on their profession or the organisation for which they work’ ( Greatbatch, 2016). Due to Health Education England CPD funding being the subject of deep cuts, and the reluctance of some organisations to release staff for CPD ( Greatbatch, 2016), it is becoming increasingly difficult for health professionals to attend courses for CPD. In order to combat the costs many universities are converting courses into e-learning ( Greatbatch, 2016).

There is increasing published research and evaluations on the effectiveness of online learning in undergraduate health professions teaching, notably nursing ( Cook *et al.,* 2008; Du *et al.*, 2013 and McCutcheon *et al.*, 2015). Yet the literature for online CPD courses is disparate. This paper attempts to provide a framework for developing online CPD courses for busy HCPs using the author’s own experiences and personal perspectives from the development and delivery of three online CPD courses and the available literature to aid future course developers. The evaluation element of course development and delivery is omitted from this paper.

## Tip 1 Before implementing a course undertake a scoping exercise and consult with stakeholders

This is the first essential, and probably the most important step is choosing an appropriate topic if you want professionals to engage in the course. Undertake a scoping exercise mapping available courses on your chosen topic and specific for your target audience. You do not want to create a course when an existing similar course is already available!

In addition to a scoping exercise, conduct a stakeholder analysis. Typically, stakeholders will include the professional body, experts in the course subject area, the hosting institution and potential participants. A combination of questionnaires (online completion and return) and if time and resources allow, focus groups can be employed to explore stakeholders opinions and perceived training needs. Try and obtain respondents from a wide range of professions, from new graduates to the experienced. Individuals with a heightened interest in the topic (i.e. they are working in the area already) may be more likely to complete the questionnaire, possibly resulting in extremes of opinion which are not representative of the entire professional population. In addition, conduct a brief assessment on potential participants’ current level of knowledge, their confidence in the proposed topic and previous training received. Findings can help narrow the course focus and inform content. Ensure this is conducted anonymously, as people tend to answer questions in a way they perceive to be socially desired or expected by the questioner ( Leung, 2001). Using these individuals as partners in the creation phase of the [course] will give an additional perspective, after all, they are your potential audience ( Pickering *et al.*, 2017).

## Tip 2 Design the course based on best pedagogical practice rather than just the technology available

As tempting as it may be to make the course fit with the technology with which you are familiar, this approach often receives criticism for its resulting overly didactic learning experiences ( Moe, 2018). ‘Findings from the literature consistently demonstrate the importance of effectively using both technology and pedagogy in the on-line environment’ ( Brinkley-Etzkom, 2018). A framework for example Mishra and Koehler’s, 2006, framework of Technological Pedagogical Content Knowledge (TPACK) needs careful consideration and application in the planning of a course design ensuring technology, pedagogy and content knowledge are not considered in isolation. Technology for learning, teaching and assessment practices should be used that is ‘based on the learning needs of students as well as the ability of teachers to adapt such technology to fit specific learning activities’ (Okojie *et al.,* n.d). In creating an online CPD course, curricular and pedagogical decisions need to be made addressing issues that are unique to eLearning, to prevent or limit their impact on the learning ( Andrews and Cole, 2015). Integrating pedagogy and technology is challenging. It is not as simple as converting face-to-face teaching materials to online format. If possible consider completing a training course on how best to apply learning technologies to teaching online, in order to become an effective online instructor/educator.

## Tip 3 Recruit tutors with experience in online learning

It cannot be assumed that course developers will be ‘digital natives’, born after 1980 and comfortable and confident participating in new technologies. Many may be ‘digital immigrants’, born before 1980 ( Margaryan, Littlejohn and Vojt, 2011) and as technology changes fast even experienced online instructors can feel like ‘perpetual novices’ ( Ertmer and Ottenbriet-Leftwich, 2010).

During the course development stage discuss your proposed course plans with experienced tutors who have successfully developed and delivered online courses. Their in-depth understanding of online learning theory and multimedia pedagogy will be equally as useful as a colleague with the course’s subject expertise. They will be able to provide valuable insights into the ‘best’ approach, based on tried and tested experience and not just upon pedagogical theory and educational principles. It would be extremely advantageous to try and find tutors who have had specific experience of developing short courses for health professionals, as the literature is sparse in this area with the majority focusing on online modules or courses with extensive learning hours. Many short courses recruit low numbers of students, are often delivered locally without thorough evaluation, and are rarely published in peer reviewed journals. It is therefore recommended that course developers search the grey literature and carry out extensive online searches to identify courses with a similar specification to their vision. Contacting the developers of a similar course could provide invaluable insights. Furthermore once you have developed your course become involved in evaluation and publication so that a body of evidence can be generated.

## Tip 4 Anticipate and give appropriate consideration for technology issues

There are common technical problems with online course delivery including ‘pop ups’ blocked, web browsers not supporting the course, the course appearing ‘stuck’ on screen and links not working.

Seek help from the learning technologist team at your institution. Their knowledge of virtual learning environments and appropriate effective media is fundamental in helping with the practicalities of developing online courses and to help ease out technical issues that may prevent student’s progressing with online learning and subsequently hindering the student experience.

Consider the resource implications of the hosting institution/organisation to be able provide IT support to efficiently respond to participants’ technical queries. From experience, it is important to ensure this support is available (ideally 24/7) when participants are accessing the course, which often is outside standard working hours (evenings and weekends) to enable their queries to be dealt with quickly (for example through a ‘Live chat’ instant messenger option), allowing them to proceed with the course.

## Tip 5 Plan learning activities considering the diverse learning needs of participants

To ensure that the course is accessible to a wide range of diverse learners, have a user centred design approach using a range of well-established multiple means of representation for teaching and learning resources for example voice over PowerPoints, videos and audio recordings.

With higher education widening entry gates, enabling individuals with less traditional educational backgrounds the opportunity to become health professionals ( Health Education England, 2014), it has contributed to an increasing proportion of students and subsequently the number of qualified health professionals with learning disabilities ( Clouder *et al*., 2016). The following strategies can help ensure your course is suitable for these participants; design learning materials using fonts that are legible on the computer, do not use colouring to convey meaning, keep the number of fonts to a minimum, avoid lengthy segments of capitalised sentences, ensure PowerPoint slides are not cluttered or frequent layout changes made from slide to slide, use solid rather than textured backgrounds, limit size and number of graphics, drawings and images and use bold or italics for emphasis rather than underlining, to avoid confusion with hyperlinks. Further, ensure that the course is inclusive by having transcripts for videos and PowerPoints for those with audio impairment.

## Tip 6 Do not neglect the social element of learning

Online learning can be an isolated ( Croft *et al*., 2010) and solitary experience for many learners.

It is important if students are going to engage in the content of the course and have a deep and meaningful learning experience that there is social, cognitive and teacher presence ( Garrison, 2009 and Salmon, 2000). To meet the learners needs it is likely the course will be ‘asynchronous’, students being able to log on to the course and work through it in their own time. This may result in there being limited chance to cultivate any learning community, as only one or two students may be logged on at any given time.

To overcome this, most online learning platforms allow the function for a discussion forum that ‘encourage affiliation and socialisation of course participants. Both foster community while creating individual comfort in a virtual class’ ( Baker, 2011). By cultivating a classroom community and stimulating intellectual discourse, students can learn from other learners engaging in the course material , who are interactively contributing knowledge and sharing experiences in purposeful critical discourse, to construct personal meaning and confirm mutual understanding i.e. a ‘community of inquiry group’ ( Garrison, 2009). This aims to encourage a deep and meaningful (collaborative-constructivist) learning experience ( Garrison, 2009) which can be used as a deliberate learning activity. Encouraging learners to communicate and collaborate in this meaningful way has been shown to be effective in enhancing learning outcomes ( Abawajy and Kim, 2011), final grade performance ( Bliuc *et al.,* 2010) and greater student perceptions of the quality and quantity of learning received ( Picciano, 2002).

At the start of a course open up the discussion forum by requesting participants to introduce themselves to other learners and ask a specific non-threatening question (e.g. tell us about the favourite part of your current role).

Despite the benefits of using a discussion forum, strategic learners need to see the value and importance of contributing to such forums. Ensure meaningful discussion tasks are designed linking to the intended course learning outcomes, content and assessment. To further engage strategic learners, include ‘active engagement’ defined as posting at least twice (e.g. responding to a question and asking a question) in the discussion forum as a requirement to pass the course. In designing the discussion forum, it is important that time is allocated for course educators to moderate and monitor the discussion. For a low number of students having a weekly one-hour diary slot allocated for this task should initially be sufficient. Depending on student numbers more than one educator and more frequent slots may be required to ensure students feel there is educator presence in the course and to demonstrate that you appreciate their contributions and to challenge them to think more deeply.

Furthermore, it would be helpful if the course developer has undertaken an online CPD course themselves to gain insight into this mode of online learning. ‘Putting yourself in the position of a learner and getting a feel for what works, and importantly what does not’ ( Pickering *et al*., 2017) may help obtain ideas that could be implemented within your own course.

## Tip 7 Ensure there is teaching presence

There is increasing literature emerging showing a strong correlation between the teaching presence of instructors and student satisfaction ( Ladyshewsky, 2013) and engagement ( Draus *et al.*, 2014).

A ‘welcome’ video by the course leader is a good starting point to ensure teaching presence is established. Research suggests that having a somewhat ‘informal’ conversational style is favourable, creating ‘a sense of social partnership’ with the course developer ‘in which learners try harder to make sense of what their conversational partner is saying’ ( Mayer, 2008). Further research has revealed that online teacher presence is a key factor related to student’s engagement and perceived learning from videos ( Hibbert, 2014).

Voice-over PowerPoints are also effective at ensuring teacher presence and adding a “talking head” shot of an instructor can make it even more engaging ( Guo, Kim and Rubin, 2014). Explore the option of using these with technology experts as many online learning platforms require students to download these files to access them on a mobile device. This additional effort may hinder engagement in the learning material.

Teacher presence can further be addressed by the course leader regularly logging into the discussion forum, responding to students’ comments, and creating announcements encouraging students to use the forum.

## Tip 8 Choose assessment methods that are meaningful and authentic

‘Assessment methods and requirements probably have a greater influence on how and what students learn than any other single factor’ ( Boud, 1988), especially for strategic learners. This remains true for online learning with assessment being identified as key in motivating engagement in learning technology interventions ( Andrews and Cole, 2015). It is important that teaching activities with accompanying ‘feasible, fair, and valid’ ( Bloxham and Boyd, 2007) assessments are designed which do not just require a recall of information, but more meaningful responses to encourage students to employ a deep learning approach ( Kember and Murphy, 1994). Online assessment methods that can achieve assesses recognition rather than recall of information include matching questions that involve matching paired lists that require students to correctly identify, or “match” depending on the relationship between the items or short answered questions.

To help determine students’ prior knowledge of the subject area, a self-assessment instrument can be used. Anonymous self-assessment instruments are effective as they are relatively easy to create and mark and with low anxiety for students. Questions on their knowledge, skills and experience can be asked through a pre-course questionnaire. These questions help students focus on the most important knowledge and skills addressed by the course and access information from prior courses or experiences that apply to the course. If prior knowledge is neglected it could result in ‘the audience learning something opposed to the educator’s intentions, no matter how well those intentions are executed’ ( Roschelle, 1995).

It is crucial that students perceive the assessment to be relevant to the working environment and see the purpose of the assessment as a useful skill transferable to their professional practice ( Reid and Fitzgerald, 2011). To make the assessments ‘authentic’ i.e. more practical, realistic and challenging ( Bloxham and Boyd, 2007), one recommendation is to contact health professionals working in the field of the course subject prior to developing the assessment and ask ‘what are the common errors that occur in that area?’ Their responses can then be used to help short answer or matching questions.

## Tip 9 Include a reflection activity

Reflection is a key skill of a health professional with a considerable emphasis upon the importance of reflection throughout their training and practice. Reflection is integral to learning, as it can help students capture and understand experiences and put their newfound knowledge into a practical context.This is essential for effective practice and can facilitate the improvement of patient care ( Jayatilleke and Mackie, 2013). Evidence shows that a reflection activity can help bring about ‘empowerment, transformative learning and outcomes that go beyond just mere acquisition of clinical knowledge online’ ( Sim and Radloff, 2008).

Careful consideration of how to engage students in a reflective activity is required, as similarly to the discussion forum, strategic learners may not complete this activity if it is not linked with assessment. Using the previously mentioned recommendation, if a reflective activity is incorporated in the discussion forum in which active engagement is required to pass the course, this can be an effective way for students to learn by sharing experiences.

## Tip 10 Determine an appropriate pass mark and have a clear disclaimer

Standard assessment pass rates are used to measure students’ learning, skills, and understanding demonstrating that they have fulfilled the objectives of the course. The virtual learning environment hosting institution may stipulate that their standard pass rate (typically 40/50%) should be applied to the online course which may be too low to determine an appropriate level of knowledge achieved for completing the course. There is a wide range of literature available on standard setting for assessments which can be employed depending on the chosen assessment method. An approach I effectively employed to determine an appropriate pass rate was to ask competent health professionals practicing in the subject field to complete the course assessment and calculate their average pass rate (70%) and adopt this for the course pass mark. The larger sample of competent health professionals you can use for this the better representation of professional practice it will be.

An online course can be used to discriminate between those who have sufficient knowledge recall and those who do not, for a particular level or purpose or subject area. Yet it is important to highlight to learners that this does not equate to clinical competency. The learning outcomes of the course should make the aims explicit, assessment information should indicate what they intend to measure (e.g. knowledge rather than skills) and include a disclaimer to clarify this on completion certificates.

Due to time restraints, electronically marked assessments (multiple choice questions) have been the chosen method for courses I have developed. If student numbers are low and course educators have sufficient capacity it may be feasible to have assessments that require manual marking (e.g. free text answers).

Whichever way assessments are marked, it is important that the correct answers are released post completion with explanations as to why the other choices were incorrect, to support learner self-regulation and provide details on which areas of the course they need to re-visit to improve their knowledge. Work with your online team/specialists to ensure you check your assessments are set up correctly to carry out this function.

## Tip 11 Carefully consider a realistic amount of time for participants to complete the course

Online learning allows students to progress at different rates. Research has shown that students need a reasonable and realistic amount of time to complete learning activities ( Kember and Murphy, 1994). Ensure material is broken down into digestible chunks, taking into consideration that the optimum length of a leaning activity is approximately 15-30 minutes ( Bennett, 2007). Apply a ‘mastery for learning’ ( Bloom, 1968) approach to the course with a systematic track release (adaptive release), the next learning material only being available once the student has clicked that they have reviewed the previous learning material. This aims to ensure students understand prerequisites for the course, foundation knowledge being learnt first before more complex topics are introduced, yet it cannot guarantee learning has occurred. Formative assessments (e.g. quizzes) could be included to help show students whether they understand the course content knowledge, and where there are gaps in their knowledge or misunderstandings that require further attention. Enabling students to be able to watch the learning materials as many times as needed will also aid ‘mastery’ of knowledge.

Although published evaluations are lacking, online CPD courses seem to follow the pattern of Massive open online courses (MOOCs) with high participant enrolment but low completion ( Onah, Sinclair and Boyatt, 2014). A recent CPD course hosted by London Metropolitan University enrolled fifty practising health professionals but only thirty percent (n=15) of participants completed the course ( Mealey *et al*., 2018). All non-completers were emailed to enquire why they chose not to move forward with the course. Seven (20%) non-completers responded. The reason for not completing the course for all these individuals was time constraint due to personal circumstances and workload. Without protected CPD time, or the CPD course being included in statutory or mandatory training intrinsic motivation is a large factor in whether an individual completes the course. When marketing the course clearly state how long it will take to complete. When piloting the course ask respondents how long it takes to complete and this will help determine a time that is attractive and achievable.

## Tip 12 Pilot the course

Piloting plays an essential role in the revision process of implementing appropriate changes to enhance learning, determining how effective the learning intervention design is and whether the different elements of the design work together. Piloting is also essential to online learning to iron out technical difficulties.

The course, including assessments should be tested before it is released to students to confirm the clarity of learning activities and to address any possible scope for misinterpretation. Try to have a diverse group of pilot learners, remembering that the online course has little ability to adapt to individual needs once developed. ‘Learning materials which are appropriate for some may take others much more (or less) time to master’ ( Onah, Sinclair and Boyatt, 2014) and thus this will help determine a realistic time to complete the learning activities.

In addition it is helpful to get an educational eLearning expert to pilot the course. They will be able to provide feedback on the consistency of the design and usability of the course, ensuring there are appropriate instructions for navigation and interaction, hyperlinks and buttons that function effectively.

Experts in the course subject should also be involved in the pilot stage to ensure content is accurate and up to date. Remember piloting and delivery of the course is the first stage and it will need ongoing review, enhancements and ensuring content is up to date and clinically relevant.

## Conclusion

The feasibility of changing from traditional face to face CPD teaching to online learning is not without challenges and students and tutors will need training and practise to feel confident with learning technology. Carefully scoping, planning and piloting with the philosophies of best pedagogical practice and seeking expertise in the field of online learning can help ensure learning activities meet the demands of busy health professionals to engage them in a supportive learning environment.

## Take Home Messages

• eLearning is feasible method to deliver short CPD courses and can have a place in education for HCPs globally
• When creating a short online CPD course attention needs to be paid to planning, carefully considering appropriate content and assessment
• Plan your course considering that the learners will have a diverse range of learning needs and approach the course in very different ways
• Collaborate with the learning technologist team at your institution to help with the practicalities of developing online courses and to ease out technical issues

## Notes On Contributors

Elaine S. Mealey BSc (Hons), MALTHE, FHEA, is a Lecturer in Clinical Communication at St. George’s University of London. She leads the communication teaching for the second-year students on the MBBS five-year program. She initiated, developed and delivered the first British Dietetic Association online course for practice educators and an eLearning module with Nestle Health Science on ‘Feeding children with neurodisability’. Her MA dissertation project involved the development and delivery of a short CPD course for dietitians on anthropometric measurements and growth monitoring in infants and children.
