# Supplementary material for: Developing an online continuing professional development course for busy healthcare professionals: 12 tips for course developers
Source: MedEdPublish (2016). 2019 Sep 12;8:170. [Version 1] doi: 10.15694/mep.2019.000170.1 (PMC10712512; doi:10.15694/mep.2019.000170.1)
Supplement: Peer review history

# Reviewer report: Reviewer response for version 1

**DOI:** 10.21956/mep.19898.r30119

**Published:** 2019-09-22

**Authors:** Helena Filipe

**Affiliations:** 1 Hospital of the Armed Forces/PL-EMGFA

This review has been migrated. The reviewer awarded 5 stars out of 5 This article focuses a relevant topic in medical education with an emphasis for the busy practicing healthcare professional: well designed and conducted online continuing professional development (CPD) activities.The article reads very well and presents a framework of a useful set of well-described twelve guidelines to assist program developers.The introduction explains the reason for the increasing CPD elearning provided by universities. I would think on clinical scenarios based emicrolearning with time allocated for peer discussion, critical self - reflection and related open elearning paths. Developing synergies and cooperation network consortiums to maximize effectiveness of CPD elearning programs is relevant.All the framework tips are fundamental to design and lead good elearning programs and each is very well described. Among others, I would emphasize the following aspects in the article:- Needs assessment based and evidence informed new programs -eLearning outcomes critically dependent on effective use of best educational strategies.-developing scholarship as a best practice -tutor led courses generally associated with fewer drop offs -the social component of learning and how it can be approached - incorporating activities to enable critical reflection - Identifying “strategic learners” as those challenging further program enhancement- establishing and sharing benchmarks with the faculty and the learnersWhen I read tips seven, eight and eleven, I respectively thought on:- synchronous interaction moments to share and discuss new insights over the didactics previously explored (flipped classroom)- assessment format - relevant content - learning outcomes.-automatized systems used in MOOC’s context to bring together learners with shared interests based on their user profiles.The conclusion is appropriate and the take home messages adequate.Thank you for the useful e most interesting article

Reviewer Expertise:

NA

No decision status is available

# Reviewer report: Reviewer response for version 1

**DOI:** 10.21956/mep.19898.r30118

**Published:** 2019-09-21

**Authors:** Mohamed Al-Eraky

**Affiliations:** 1 University of Dammam

This review has been migrated. The reviewer awarded 4 stars out of 5 Thank you for this interesting article that addressed a common problem on how to plan and execute CPD for ‘busy’ clinicians. The tips are narrated in a logical manner and provides a structured plan to course developers. With respect to needs assessment, we used to seek inputs and opinions from various stakeholders. It’s also important to consider other inputs in planning, such as: baseline performance (competency), mission of the institution and the availability of resources. If wishes from potential participants can be considered one input, e.g. West. These can be considered East, North and South directions to the plan, as indicated in the Compass Model in this link:https://drive.google.com/open?id=0ByugEmLvxFlUaGQydTBBX0h2czQ For online courses, I suggest reviewing the standards of international authorities like: Quality Matters. https://www.qualitymatters.org As they provide guidelines and further assistance to develop e-courses that work. Thank you.

Reviewer Expertise:

NA

No decision status is available
